# Carbohydrate antigen 125, carbohydrate antigen 15–3 and low-density lipoprotein as risk factors for intraocular metastases in postmenopausal breast cancer

**DOI:** 10.1097/MD.0000000000027693

**Published:** 2021-10-29

**Authors:** Jing Tang, Bo Yan, Gao-Feng Li, Qiu-Yu Li, Wen-Feng Liu, Rong-Bin Liang, Qian-Min Ge, Yi Shao

**Affiliations:** a Department of Oncology, the Affiliated Zhuzhou Hospital Xiangya Medical College CSU, Zhuzhou, Hunan, China; b Hunan University of Technology, Zhuzhou, Hunan, China; c Department of Ophthalmology, the First Affiliated Hospital of Nanchang University, Nanchang, Jiangxi Province, China; d Liver Cancer Institute, Zhongshan Hospital, Fudan University, Shanghai, China.

**Keywords:** carbohydrate antigen 15–3, carbohydrate antigen-125, intraocular metastases, low-density lipoprotein, postmenopausal breast cancer

## Abstract

The prognosis of patients with postmenopausal breast cancer (PBC) could be improved by the early detection of intraocular metastases (IOMs). However, serum biomarkers for IOMs in PBC remain elusive. In the current study, we investigated patients with PBC, and compared serum parameters in an IOM and a non-IOM group, and then differentiated the risk factors related to IOMs. A comparison between an IOM and a non-IOM (NIOM) group was performed using Student *t*-test and a Chi-Squared test. After constructing a Poisson regression model to identify risk factors, we plotted receiver operating characteristic curves to evaluate the predictive value of significant risk factors in detecting IOMs. The incidence of IOMs in PBC was 1.16%. The histopathology results were not significantly different between the 2 groups. The levels of serum carbohydrate antigen 125 (CA-125), carbohydrate antigen 15–3 (CA15–3) and alkaline phosphatase were significantly elevated in IOMs compared with NIOMs (*P* = .082, *P* < .001, and *P* < .001, respectively). Compared with NIOMs, age, carbohydrate antigen 19 to 9, hemoglobin, calcium, total cholesterol, low-density lipoprotein (LDL) and apolipoprotein A1 were remarkably lower in IOMs (*P* = .038, *P* < .001, *P* < .001, *P* = .032, *P* = .041, *P* < .001, and *P* = .001, respectively). Poisson regression suggested that CA-125, CA15–3 and LDL were contributing to IOMs in PBC as risk factors (OR = 1.003, 95% CI: 1.001–1.005; OR = 1.025, 95% CI: 1.019–1.033; OR = 0.238, 95% CI: 0.112–0.505, respectively). A receiver operating characteristic curve revealed that the cut-off values for CA-125, CA15–3 and LDL were 16.78 0 U/mL, 63.175 U/mL, and 2.415 mmol/L, respectively. The combination of CA-125 and CA15–3 showed significant diagnostic value (area under the curve [AUC] = 0.982, *P* < .001). Our investigation suggests that CA-125, CA15–3 and LDL remarkably predict IOMs in PBC as risk factors, and the combination of CA-125 and CA15–3 shows considerable diagnostic value.

## 1 Introduction

Breast cancer (BC) is the most common cancer in females world-wide, and BC is the main source of cancer-related death in women.^[1]^ Metastases play an important role in cancer-related death, and improvements in therapies for metastatic cancer have been slow to emerge.^[2]^ Currently, to facilitate postmenopausal BC (PBC) prognosis, identifying early metastases is necessary.

Intraocular metastases (IOMs) are regarded as a leading cause of intraocular malignancy.^[3]^ The choroid is the most common site of IOMs.^[4]^ It was reported in a cohort study involving 1111 participants, that BC demonstrated the highest proportion of IOMs, at 37%, and the average age at which patients experienced IOMs was 57.^[5]^ In other words, despite the low incidence of IOMs in BC, BC is the main primary lesion for IOMs.

Currently, imaging techniques are indispensable in detecting ocular tumors, and these include computed tomography (CT), high-frequency ultrasound, fluorescein angiography optical coherence tomography, and magnetic resonance imaging (MRI). ^[6]^ However, the cost and radiation dose associated with such techniques are too high for long-term follow-ups. Moreover, the incidence of IOMs is somewhat low. Consequently, although these imaging tests have considerable diagnostic potential in this context, they are not suitable as a global-scale tool to regularly screen PBC patients during long-term follow-up. However, serological monitoring is non-invasive, reproducible, and cost-effective, which make it a better option for detecting IOMs.^[7]^ Recently, it has been confirmed that serum monitoring has considerable potential in BC diagnosis and screening; for instance, monitoring of exosomal miRNA^[8]^ and serum 25-hydroxyvitamin D^[9]^ has been shown to be useful. To promote the ability of early detecting IOMs in PBC, the identification of serum risk factors is necessary and is becoming more feasible.

With the goal of improving PBC patient prognosis, a retrospective study was conducted by our research group to identify serum risk factors for IOMs in PBC by evaluating the link between clinical serum pathological parameters and IOMs.

## 2 Materials and methods

### 2.1 Ethics statement

The Medical Research Ethics Committee of the First Affiliated Hospital of Nanchang University approved this study (CDYFY-LL2006023). Because of the retrospective nature of the study, informed consent of participants was waived. The methodology used in this study is in accordance with approved guidelines and related regulations.

### 2.2 Study design

Participants with BC involved in this retrospective observational study from April 1995 to July 2017 were diagnosed via histopathological sections obtained through surgical resection or biopsy. The IOMs were confirmed through local CT and MRI. The key inclusion criteria were female BC patients with postmenopausal status, and BC was the primary lesion. The key exclusion criteria were patients with primary ocular malignancies or benign tumors without pathology reports.

### 2.3 Data collection

The patient medical records were the source of the study data. All data, such as demographic characteristics and clinical pathology parameters, were collected before patients received anti-tumor therapy (radiotherapy, chemotherapy, or surgery). These mainly included: the age of the tumor being diagnosed, histopathology and the condition of metastases, and laboratory results such as alkaline phosphate, hemoglobin, triglycerides, low-density lipoprotein (LDL), apolipoprotein A, and serum calcium levels. The concentrations of common tumor markers in serum, such as carcinoembryonic antigen, cancer antigen 125 (CA-125), cancer antigen 153 (CA15–3) and cancer antigen 19–9, were also recorded. As the link between different serological pathological parameters and IOMs was analyzed, we estimated the predictive value of significant risk factors in detecting IOMs.

### 2.4 Statistical analyzes

SPSS software (version 19.00; SPSS, Chicago, IL) and GraphPad Prism (8.0.1.244) were used for statistical analysis, and Excel 2010 (Excel, Microsoft Corp, USA) was used for data collation. Initially, a comparison between an IOM and a non-IOM (NIOM) group was performed using Student *t* test and the Chi-Squared test to find clinical pathology parameters with significant differences. Quantitative variables are reported as mean ± standard deviation. The occurrence of IOMs in PBC is a rare event (10/865; ≈0.0116). Consequently, a multivariate Poisson regression analysis model for rare events was constructed to identify statistically independent risk factors and determine the odds ratio (OR) and 95% confidence interval (95% CI). Finally, receiver operating characteristic (ROC) curves were plotted to evaluate the predictive value of significant risk factors in detecting IOMs. The area under the curve (AUC) of the different factors, and the sensitivity and specificity of the optimal cut-off point were calculated to estimate the capability of the risk factors in predicting IOMs. *P* values ofless than 0.05 were regarded as statistically significant, and all reported *P* values were bilateral. When adding variables into the Poisson regression analysis model, we used *P* < .20 as a standard to ensure that low-impact factors were not being missed. The test level (α) was set to 0.05.

## 3 Results

### 3.1 Demographics and clinical characteristics

A total of 2373 PBC patients participated in this investigation, and 865 patients were finally involved based on the inclusion and exclusion criteria. Table 1 lists baseline data such as the demographic and clinical characteristics of the IOM group (10 patients) and the NIOM group (855 patients). As for age, we observed that patients in the IOMs were much older than that in the NIOMs (*P* = .038). The histopathology results were not significantly different between the 2 groups. Besides, most IOMs were 2 to 5 cm and TNM 2–3 Stage. Detailed clinical data for all elderly patients participating in the study are listed in Table 1.

**Table 1:** The clinical characteristics of patients with PBC.

Characteristics	Total number of patients (%)	IOM group (n = 10)	NIOM group (n = 855)	*P*^∗^ value
Age (yr)^a^	58.24±7.77	63.30±3.86	58.18±7.78	**.038**
Histopathology(n)^b^				.406
Invasive ductal carcinoma	429	6	423	
Other types	393	4	389	
Unknown	43	0	43	
Tumor size(cm)^b^				**.020**
<2	65	1	64	
2–5	522	9	513	
>5	0	0	0	
Unkonwn	278	0	278	
Stage^b^				**.001**
I	37	0	37	
II	400	3	397	
III	144	7	137	
IV	37	0	37	
Unkonwn	247	0	247	

### 3.2 Differences in the clinical features and the risk factors associated with intraocular metastases

The results showed that triglyceride, high density lipoprotein, apolipoprotein B, lipoprotein a and carcino-embryonic antigen were not significantly different between the IOM and NIOM group (*P* > .05). However, serum CA-125, CA15–3 and alkaline phosphatase levels were significantly elevated in the IOM group compared with the NIOM group (*P* = .082, *P* < .001, and *P* < .001, respectively). Compared with the NIOM group, age, CA-19–9, hemoglobin, calcium, total cholesterol, LDL and apolipoprotein A1 were lower in the IOM group (*P* = .038, *P* < .001, *P* < .001, *P* = .032, *P* = .041, *P* < .001 and *P* = .001, respectively) (Table 2). The Poisson regression model showed that CA-125, CA15–3 and LDL were contributing to IOMs in PBC as risk factors (OR = 1.003, 95% CI: 1.001–1.005; OR = 1.025, 95% CI: 1.019–1.033; OR = 0.238, 95% CI: 0.112–0.505, respectively) (Table 3).

**Table 2:** The differences of clinical characteristics between patients with and without IOMs.

Characteristics	IOMs group	NIOMs group	*T* value	*P* value^∗^
Age(yr)	63.30 ± 3.86	58.18 ± 7.78	2.076	.038
CEA(ng/mL)	31.89 ± 62.09	14.80 ± 277.91	0.782	.447
CA-125(U /mL)	114.84 ± 171.62	21.86 ± 167.86	1.741	.082
CA15–3(U /mL)	137.70 ± 61.42	19.66 ± 34.73	10.569	<.001
CA19–9(U /mL)	7.82 ± 3.82	16.06 ± 24.95	−5.56	<.001
HB(g/L)	101.20 ± 9.47	122.43 ± 13.46	−7.005	<.001
ALP(U/L)	120.20 ± 58.77	78.97 ± 35.83	3.586	<.001
Ca(mmol / L)	2.10 ± 0.49	2.50 ± 0.53	−2.516	.032
TC (mmol/L)	4.89 ± 1.15	5.79 ± 4.46	−2.277	.041
TG(mmol/L)	1.58 ± 0.63	2.34 ± 1.85	−1.307	.192
HDL (mmol/L)	2.26 ± 1.78	2.24 ± 1.73	0.033	.975
LDL (mmol/L)	1.73 ± 0.89	3.66 ± 1.75	−6.673	<.001
APOA1(g/L)	1.06 ± 0.43	1.70 ± 0.44	−4.654	.001
ApoB (g/L)	1.31 ± 0.45	1.61 ± 1.30	−0.732	.464
Lp(a)(mg/L)	223.50 ± 121.20	193.55 ± 207.25	0.768	.461

**Table 3:** The poisson regression results.

Factors	B	OR	OR (95% CI)	*P* value^∗^
Age	0.027	1.027	0.948–1.113	.505
CA-125	0.003	1.003	1.001–1.005	.016
CA15–3	0.025	1.025	1.019–1.033	<.001
CA19–9	−0.163	0.850	0.695–1.040	.114
HB	−0.021	0.979	0.943–1.016	. 268
lALP	−0.008	0.992	0.983–1.002	.128
Ca	−1.679	0.187	0.020–1.735	.140
TC	0.027	1.027	0.990–1.066	.147
TG	−0.285	0.752	0.468–1.209	.240
LDL	−1.437	0.238	0.112–0.505	<.001
APO-A1	−1.868	0.154	0.007–3.421	.237

### 3.3 The cut-of value, area under the curve, sensitivity, and specificity of carbohydrate antigen 125, carbohydrate antigen 15–3, and low-density lipoprotein in diagnosing intraocular metastases

**Figure 1:**
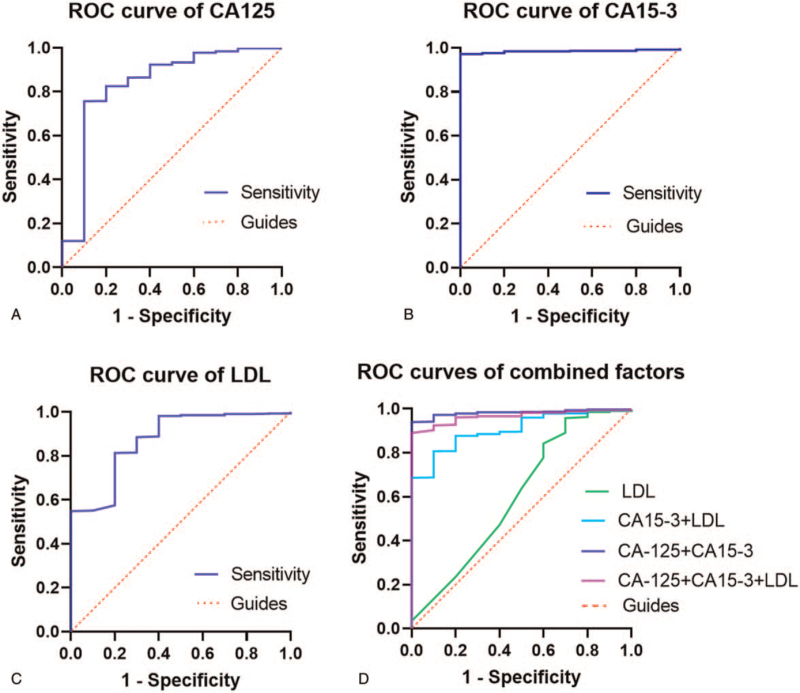
The ROC curves of risk factors for IOMs in PBC. (A) The ROC curve of CA-125. The AUC was 0.838 (*P* value <.001; 95% CI: 0.6822–0.9945) (IOMs>NIOMs). (B) The ROC curve of CA15–3. The AUC was 0.984 (*P* value <.001; 95% CI: 0.9760–0.9927) (IOMs>NIOMs). (C) The ROC curve of LDL. The AUC was 0.874 (*P* value <.001; 95% CI: 0.7692–0.9795) (IOMs<NIOMs). (D) The ROC curve of a combination of CA-125 and CA15–3. The AUC was 0.982 (*P* value <.001; 95% CI: 0.9704–0.9943). The ROC curve of a combination of CA-125 and LDL. The AUC was 0.604 (*P* value = .256; 95% CI: 0.3991–0.8096). The ROC curve of a combination of CA-153 and LDL. The AUC was 0.906 (*P* value <.001; 95% CI: 0.8462–0.9654). The ROC curve of a combination of CA-125, CA-153 and LDL. The AUC was 0.965 (*P* value <.001; 95% CI: 0.9451–0.9867). ROC = receiver operating characteristic, AUC = area under the curve, CI = confidence interval, IOMs = intraocular metastases, NIOMs = non-intraocular metastases.

The ROC curve showed that CA15–3 had the highest predictive accuracy for IOMs (AUC = 0.984), and its sensitivity and specificity was 100.0% and 97.2%, respectively (Table 4 and Fig. 1). The cut-off values for CA-125, CA15–3 and LDL were 16.78 0 U/mL, 63.175 U/mL, and 2.415 mmol/L, respectively. In other words, PBC patients with CA-125 >16.78 0 U/mL, CA15–3 >63.175 U/mL, and LDL < 2.415 mmol/L are at greater risk of IOMs. The results for the combined risk factors showed that a combination of CA-125 and CA15–3 had a higher diagnostic accuracy for IOMs than the single factors (AUC = 0.982, *P* < .001) (Table 4 and Fig. 1).

**Table 4:** The ROC results of risk factors for predicting IOMs in breast cancer patients.

Factors	Cut-off value	Sensitivity (%)	Specificity (%)	AUC	*P* value^∗^
CA-125 (U/mL)	16.78	90.0	75.7	0.838	<.001
CA15–3 (U/mL)	63.175	100.0	97.2	0.984	<.001
LDL (mmol/L)	2.415	81.4	80.0	0.874	<.001
CA-125+ CA15–3	–	100.0	94.1	0.982	<.001
CA-125+ LDL	–	40.0	87.8	0.604	.256
CA15–3+LDL	–	90.0	80.8	0.906	<.001
CA-125+CA15–3+ LDL		100.0	89.2	0.965	<.001

## 4 Discussion

The incidence of IOMs in BC is low, at approximately 0.07% to 12%.^[10]^ In the current study, the incidence of IOMs in PBC was 1.16%, within the range of 0.07% to 12%. We also found that patients in the IOMs were much older than that in the NIOMs. However, the result of poisson regression showed that p value of ages was 0.505, indicating there was no significant correlation between ages and IOMs. It may because the number of patients in IOMs group was limited. Besides, most IOMs were 2 to 5 cm and TNM 2–3 Stage, indicating IOM can occur at an early stage of PBC.

**Figure 2:**
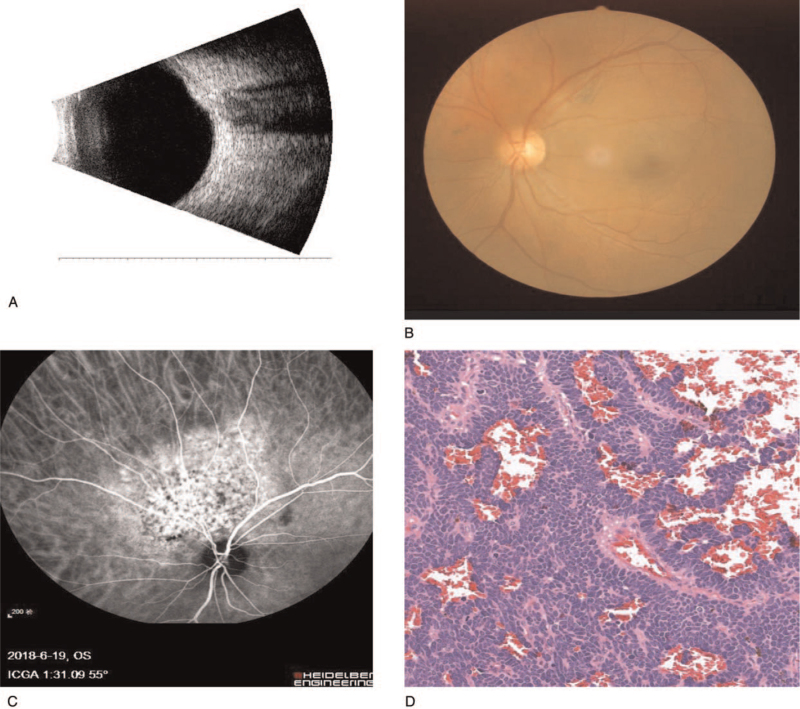
Examples of IOMs. (A) B-ultrasound of an eye with IOMs (left eye): A solid echo of a strong echo is visible in the posterior pole, and the mass grows along the wall of the ball. (B) Fundus photography an eye with IOMs (right eye): A yellow-white, nodular flat bulge can be seen under the retina of the posterior pole. (C) Fundus angiography of an eye with IOMs (right eye): Fluorescence leakage lesion, high fluorescence, and angiography in the lesion are visible. (D) Pathological images of IOMs HE stain × 200. IOMs = intraocular metastases; HE = hematoxylin and eosin.

The most frequent symptoms of IOMs are blurred vision and sight loss, which are non-specific symptoms, and the prognosis for IOMs is poor.^[11]^ Moreover, according to a case report, after 34 years of BC remission, IOMs occurred.^[12]^ Therefore, there is a necessity for long-term screening for IOMs. However, adherence to follow-up visits is low in PBC.^[13]^ Consequently, the management of IOMs in PBC is confronted with an enormous challenge, because of non-specific IOM symptoms, unfavorable adherence, excessive follow-up times and the low incidence of IOMs. Moreover, imaging tests (Fig. 2) are likely not suitable to regularly screen PBC patients during long-term follow-up. However, recently, it has been revealed that serum biomarkers are related to prognosis in many cancers, suggesting that serological monitoring will play an increasingly important role in the detection of cancer during follow-up (Table 5). Therefore, to permit the early detection of IOMs in PBC, the identification of serum risk factors is necessary and is becoming more feasible.

**Table 5:** The serum tumor biomarkers as risk factors of different types of cancer.

Author	Year	Cancer	Serum tumor biomarkers
Guida F^[17]^	2018	Lung cancer	CA-125,CEA, CYFRA 21–1 Pro-SFTPB
Luo G ^[18]^	2017	Pancreatic cancer	CEA, CA-125
Namikawa T^[19]^	2018	Gastric cancer	CA-125
Gao Y^[20]^	2018	Colorectal cancer	CEA, CA19–9, CA72–4, CA-125, Ferritin
Imai K^[21]^	2016	Endometrial cancer	CA-125
Gupta D^[22]^	2011	uterine papillary Serous Carcinoma	CA-125
Molina R^[28]^	2016	Lung cancer	CEA, CA15–3, SCC, CYFRA 21–1, NSE, Pro-GRP
Li J^[29]^	2013	Cervical cancer	CA15–3, TNF-α
Zhang B^[30]^	2016	Colorectal cancer	CA15–3, CA-125, CA19–9, CA242
Hong TT^[42]^	2016	Colorectal cancer	LDL-C, TC, TG, HDL-C
Zhang GM^[43]^	2015	Prostate cancer	LDL, TC, TG
Deng H^[44]^	2019	Esophageal squamous cell carcinoma	LDL
McCaw L^[45]^	2017	Chronic lymphocytic leukemia	LDL

CA-125, known as Mucin 16, is the largest mucin, and its overexpression occurs in numerous cancers.^[14]^ It was revealed by a multicenter study that CA-125 has considerable diagnostic value in epithelial ovarian cancers (EOCs).^[15]^ CA-125 exerts an important role in EOC cell proliferation and metastases.^[16]^ Additionally, CA-125 has been linked to lung cance, pancreatic cancer, gastric cancer, colorectal cancer, endometrial cancer, and uterine papillary serous carcinoma.^[17–22]^ It was also reported that CA-125 shows diagnostic value in identifying BC recurrence.^[23]^ Moreover, it was confirmed that CA-125 has considerable value in diagnosing metastatic BC.^[24]^ A study involving 2133 BC patients revealed that CA-125 was related to bone metastases in BC.^[25]^ In fact, the *P* value of CA-125, (IOM vs NIOM) was 0.082 (Table 2), which was more than 0.05 and not statistically significant in the Student *t* test. In the Poisson regression analysis model, we used *P* < .20 as a standard to ensure that low-impact factors were not being missed and the OR of CA-125 turned out to be significant (*P* = .016, Table 3). Therefore, we hypothesized that CA-125 is a risk factor for IOMs in PBC. The current study revealed that the occurrence of IOMs is more likely in PBC patients with CA-125 >16.78 0 U/mL.

CA15–3, also known as Mucin 1, is a membrane tethered glycoprotein, which contributes to cancer progression.^[26]^ It was reported in a meta-analysis that CA15–3 could be a cancer biomarker.^[27]^ Moreover, numerous studies have reported that CA15–3 is relevant in lung cancer, cervical cancer and colorectal cancer, and it is also useful in the diagnosis of malignant pleural effusion.^[28–31]^ It was demonstrated by a study involving 1681 participants that CA15–3 in high levels plays a crucial role in the BC tumor burden.^[32]^ Serum levels of CA15–3 are recommended for use in detecting metastatic BC by the American Society of Clinical Oncology Clinical Practice Guidelines.^[33]^ It was reported by Wu SG et al^[34]^ that CA15–3 is a risk factor for axillary lymph node metastasis in BC. Similarly, this link was also observed for liver metastasis in BC.^[35]^ In a recent case report it was observed that CA15–3 increased in a 57-year-old BC patient with IOMs that arose 28 years after the initial BC diagnosis. ^[36]^ Consequently, we identified CA15–3 as a risk factor for IOMs in PBC. Patients with CA15–3 >63.175 U/mL are more likely to suffer IOMs.

LDL, a lipoprotein granule abundant in cholesterol, transports cholesterol to peripheral tissues from the liver.^[37]^ LDL has been correlated with ocular diseases such as meibomian gland dysfunction and age-related cataracts.^[38,39]^ Recently, it has been demonstrated that metabolic dysregulation contributes to an elevation in cancer mortality.^[40]^ It was reported by Guang et al that LDL has an adverse link with the total risk of cancer.^[41]^ Serum LDL has been linked to colorectal cancer, prostate cancer, esophageal squamous cell carcinoma and chronic lymphocytic leukemia. ^[42–45]^ LDL has been reported as a risk factor in the context of cancer progression and metastases.^[46]^ Moreover, it was reported by Kumar et al. that high levels of LDL are linked to BC.^[47]^ Increased BC proliferation and metastasis was observed following LDL stimulation, as the levels of tumor LDL receptors increased.^[48]^ However, it was demonstrated that BC patients with metastases had a significantly lower level of LDL compared with a control group.^[49]^ This may be because of the large consumption of LDL during rapid proliferation and metastases in BC. Therefore, we hypothesized that LDL at a low level was a risk factor for IOMs in PBC. The current study demonstrated that patients with LDL <2.415 mmol/L are at greater risk for IOMs. However, the ROC results indicated that the accuracy of LDL in predicting IOMs is not sufficient to detect IOMs alone.

Although the results are promising, the current study still has some limitations. Firstly, this is a retrospective observational study. As the study period is substantial, some correlative data are not adequate, such as patient survival time. Moreover, there may be differences in the methodological and technical aspects within the assay of tumor markers and biochemical parameters, which may exert a negative effect on the sensitivity and specificity of test parameters. Besides, the sample size of IOMs is limited and it may not be sufficiently large to enable the extrapolation of the results to the entire BC population. Thirdly, some confounding bias may be present, because all of the data in the current study derived from a single medical institution. Therefore, the results of the current study need to be validated through a multicenter study.

## 5 Conclusion

To summarize, this investigation revealed that CA-125, CA15–3, and LDL have considerable predictive value for IOMs in PBC as risk factors. These markers will facilitate the early detection of IOMs in PBC during long-term follow-up. Moreover, these results could inspire novel insights into the molecular mechanisms of CA-125, CA15–3, and LDL for antineoplastic applications.

## Author contributions

**Conceptualization:** Jing Tang, Bo Yan, Gao-Feng Li.

**Data curation:** Jing Tang, Bo Yan, Gao-Feng Li.

**Formal analysis:** Jing Tang, Bo Yan, Gao-Feng Li.

**Investigation:** Qiu-Yu Li.

**Methodology:** Wen-Feng Liu.

**Project administration:** Jing Tang and Bo Yan.

**Supervision:** Qian-Min Ge, Yi Shao.

**Validation:** Wen-Feng Liu.

**Writing – original draft:** Jing Tang, Bo Yan, Gao-Feng Li.

**Writing – review & editing:** Jing Tang, Bo Yan, Rong-Bin Liang, Yi Shao.

## References

[1] Bray F Ferlay J Soerjomataram I. Global cancer statistics 2018: GLOBOCAN estimates of incidence and mortality worldwide for 36 cancers in 185 countries. CA Cancer J Clin 2018;68:394–424. 30207593 10.3322/caac.21492

[2] Riggi N Aguet M Stamenkovic I. Cancer metastasis: a reappraisal of its underlying mechanisms and their relevance to treatment. Annu Rev Pathol 2018;13:117–40. 29068753 10.1146/annurev-pathol-020117-044127

[3] Chen CJ McCoy AN Brahmer J. Emerging treatments for choroidal metastases. Surv Ophthalmol 2011;56:511–21. 22117885 10.1016/j.survophthal.2011.05.001 PMC4732700

[4] Mathis T Jardel P Loria O. New concepts in the diagnosis and management of choroidal metastases. Prog Retin Eye Res 2019;68:144–76. 30240895 10.1016/j.preteyeres.2018.09.003

[5] Shields CL Welch RJ Malik K. Uveal metastasis: clinical features and survival outcome of 2214 tumors in 1111 patients based on primary tumor origin. Middle East Afr J Ophthalmol 2018;25:81–90. 30122853 10.4103/meajo.MEAJO_6_18 PMC6071342

[6] Maheshwari A Finger PT. Cancers of the eye. Cancer Metastasis Rev 2018;37:677–90. 30203109 10.1007/s10555-018-9762-9

[7] suchiya N Sawada Y Endo I. Biomarkers for the early diagnosis of hepatocellular carcinoma. World J Gastroenterol 2015;21:10573–83. 26457017 10.3748/wjg.v21.i37.10573 PMC4588079

[8] Rodríguez-Martínez A de Miguel-Pérez D Ortega FG. Exosomal miRNA profile as complementary tool in the diagnostic and prediction of treatment response in localized breast cancer under neoadjuvant chemotherapy. Breast Cancer Res 2019;21:21 doi: 10.1186/s13058-019-1109-0. 30728048 10.1186/s13058-019-1109-0 PMC6366103

[9] McDonnell SL Baggerly CA French CB. Breast cancer risk markedly lower with serum 25-hydroxyvitamin D concentrations >/=60 vs <20 ng/mL (150 vs 50 nmol/L): pooled analysis of two randomized trials and a prospective cohort. PLoS One 2018;13:e0199265 doi: 10.1371/journal.pone.0199265. 29906273 10.1371/journal.pone.0199265 PMC6003691

[10] Rishi P Dixit A Verma A. Bilateral optic disk metastasis from breast carcinoma. Indian J Ophthalmol 2015;63:451–2. 26139809 10.4103/0301-4738.159886 PMC4501144

[11] Jardel P Sauerwein W Olivier T. Management of choroidal metastases. Cancer Treat Rev 2014;40:1119–28. 25451606 10.1016/j.ctrv.2014.09.006

[12] Randhawa S Johnson RN. Choroidal metastases 34 years after remission of breast cancer. Retin Cases Brief Rep 2015;9:25–9. 25383858 10.1097/ICB.0000000000000069

[13] Ziller V Kalder M Albert US. Adherence to adjuvant endocrine therapy in postmenopausal women with breast cancer. Ann Oncol Off J Eur Soc Med Oncol 2009;20:431–6. 10.1093/annonc/mdn646 19150950

[14] Aithal A Rauth S Kshirsagar P. MUC16 as a novel target for cancer therapy. Expert Opin Ther Targets 2018;22:675–86. 29999426 10.1080/14728222.2018.1498845 PMC6300140

[15] Romagnolo C Leon AE Fabricio ASC. HE4, CA125 and risk of ovarian malignancy algorithm (ROMA) as diagnostic tools for ovarian cancer in patients with a pelvic mass: an Italian multicenter study. Gynecol Oncol 2016;141:303–11. 26801941 10.1016/j.ygyno.2016.01.016

[16] Theriault C Pinard M Comamala M. MUC16 (CA125) regulates epithelial ovarian cancer cell growth, tumorigenesis and metastasis. Gynecol Oncol 2011;121:434–43. 21421261 10.1016/j.ygyno.2011.02.020

[17] Guida F Sun N Bantis LE. Assessment of lung cancer risk on the basis of a biomarker panel of circulating proteins. JAMA Oncol 2018;4:e182078 doi: 10.1001/jamaoncol.2018.2078. 30003238 10.1001/jamaoncol.2018.2078 PMC6233784

[18] Luo G Liu C Guo M. Potential biomarkers in lewis negative patients with pancreatic cancer. Ann Surg 2017;265:800–5. 28267695 10.1097/SLA.0000000000001741

[19] Namikawa T Kawanishi Y Fujisawa K. Serum carbohydrate antigen 125 is a significant prognostic marker in patients with unresectable advanced or recurrent gastric cancer. Surg Today 2018;48:388–94. 29043453 10.1007/s00595-017-1598-3

[20] Gao Y Wang J Zhou Y. Evaluation of serum CEA, CA19-9, CA72-4, CA125 and ferritin as diagnostic markers and factors of clinical parameters for colorectal cancer. Sci Rep 2018;8:2732 doi: 10.1038/s41598-018-21048-y. 29426902 10.1038/s41598-018-21048-y PMC5807317

[21] Imai K Kato H Katayama K. A preoperative risk-scoring system to predict lymph node metastasis in endometrial cancer and stratify patients for lymphadenectomy. Gynecol Oncol 2016;142:273–7. 27268220 10.1016/j.ygyno.2016.06.004

[22] Gupta D Gunter MJ Yang K. Performance of serum CA125 as a prognostic biomarker in patients with uterine papillary serous carcinoma. Int J Gynecol Cancer 2011;21:529–34. 21436701 10.1097/IGC.0b013e31821091b5

[23] Li L Gao Q Xu G. Postoperative recurrence analysis of breast cancer patients based on clinical serum markers using discriminant methods. Cancer Biomark 2017;19:403–9. 28582844 10.3233/CBM-160322 PMC13020747

[24] Wang W Xu X Tian B. The diagnostic value of serum tumor markers CEA, CA19-9, CA125, CA15-3, and TPS in metastatic breast cancer. Clin Chim Acta 2017;470:51–5. 28457854 10.1016/j.cca.2017.04.023

[25] Chen WZ Shen JF Zhou Y. Clinical characteristics and risk factors for developing bone metastases in patients with breast cancer. Sci Rep 2017;7:11325 doi: 10.1038/s41598-017-11700-4. 28900285 10.1038/s41598-017-11700-4 PMC5595860

[26] Nath S Mukherjee P. MUC1: a multifaceted oncoprotein with a key role in cancer progression. Trends Mol Med 2014;20:332–42. 24667139 10.1016/j.molmed.2014.02.007 PMC5500204

[27] Li X Xu Y Zhang L. Serum CA153 as biomarker for cancer and noncancer diseases. Prog Mol Biol Transl Sci 2019;162:265–76. 30905456 10.1016/bs.pmbts.2019.01.005

[28] Molina R Marrades RM Auge JM. Assessment of a combined panel of six serum tumor markers for lung cancer. Am J Respir Crit Care Med 2016;193:427–37. 26465739 10.1164/rccm.201404-0603OC

[29] Li J Cheng H Zhang P. Prognostic value of combined serum biomarkers in predicting outcomes in cervical cancer patients. Clin Chim Acta 2013;424:292–7. 23850705 10.1016/j.cca.2013.07.003

[30] Zhang B Liang XL Gao HY. Models of logistic regression analysis, support vector machine, and back-propagation neural network based on serum tumor markers in colorectal cancer diagnosis. Genet Mol Res 2016;15:01–10. 10.4238/gmr.15028643 27323037

[31] Wang XF Wu YH Wang MS. CEA, AFP, CA125, CA153 and CA199 in malignant pleural effusions predict the cause. Asian Pac J Cancer Prev 2014;15:363–8. 24528057 10.7314/apjcp.2014.15.1.363

[32] Lee JS Park S Park JM. Elevated levels of preoperative CA 15-3 and CEA serum levels have independently poor prognostic significance in breast cancer. Ann Oncol Off J Eur Soc Med Oncol 2013;24:1225–31. 10.1093/annonc/mds604 23230137

[33] Van Poznak C Somerfield MR Bast RC. Use of biomarkers to guide decisions on systemic therapy for women with metastatic breast cancer: American society of clinical oncology clinical practice guideline. J Clin Oncol 2015;33:2695–704. 26195705 10.1200/JCO.2015.61.1459 PMC5478102

[34] Wu SG He ZY Ren HY. Use of CEA and CA15-3 to predict axillary lymph node metastasis in patients with breast cancer. J Cancer 2016;7:37–41. 26722358 10.7150/jca.13090 PMC4679379

[35] Cao R Wang LP. Serological diagnosis of liver metastasis in patients with breast cancer. Cancer Biol Med 2012;9:57–62. 23691457 10.3969/j.issn.2095-3941.2012.01.011 PMC3643646

[36] Luo Z Cai Q Zhao Y. Late distant recurrence of breast carcinoma and metastasis to the main bronchus and choroid: a case report. Medicine (Baltimore) 2018;97:e10754 doi: 10.1097/MD.0000000000010754. 29768356 10.1097/MD.0000000000010754 PMC5976331

[37] Guan X Liu Z Zhao Z. Emerging roles of low-density lipoprotein in the development and treatment of breast cancer. Lipids Health Dis 2019;18:137 doi: 10.1186/s12944-019-1075-7. 31182104 10.1186/s12944-019-1075-7 PMC6558919

[38] Osae EA Steven P Redfern R. Dyslipidemia and meibomian gland dysfunction: Utility of lipidomics and experimental prospects with a diet-induced obesity mouse model. Int J Mol Sci 2019;20:3505 doi: 10.3390/ijms20143505. 10.3390/ijms20143505 PMC6678820 31319467

[39] Li S Li D Zhang Y. Association between serum lipids concentration and patients with age-related cataract in China: a cross-sectional, case-control study. BMJ Open 2018;8:e021496 doi: 10.1136/bmjopen -2018-021496. 10.1136/bmjopen-2018-021496 PMC5892756 29626052

[40] Dibaba DT Judd SE Gilchrist SC. Association between obesity and biomarkers of inflammation and metabolism with cancer mortality in a prospective cohort study. Metabolism 2019;94:69–76. 30802456 10.1016/j.metabol.2019.01.007 PMC7401298

[41] Guan XM Wu SL Yang XL. Association of total cholesterol, low-density lipoprotein cholesterol, and non-high-density lipoprotein cholesterol with atherosclerotic cardiovascular disease and cancer in a Chinese male population. Int J cancer 2018;142:1209–17. 29119548 10.1002/ijc.31149

[42] Hong TT Shen D Chen XP. Preoperative serum lipid profile and outcome in nonmetastatic colorectal cancer. Chronic Dis Transl Med 2016;2:241–9. 29063049 10.1016/j.cdtm.2016.11.015 PMC5643756

[43] Zhang GM Qin XJ Zhang HL. Serum lipid profiles: novel biomarkers predicting advanced prostate cancer in patients receiving radical prostatectomy. Asian J Androl 2015;17:239–44. 25475662 10.4103/1008-682X.142135 PMC4650485

[44] Deng H Zhou T Mo X. Low-density lipoprotein promotes lymphatic metastasis of esophageal squamous cell carcinoma and is an adverse prognostic factor. Oncol Lett 2019;17:1053–61. 30655865 10.3892/ol.2018.9683 PMC6313071

[45] McCaw L Shi Y Wang G. Low density lipoproteins amplify cytokine-signaling in chronic lymphocytic leukemia cells. EBioMedicine 2017;15:24–35. 27932296 10.1016/j.ebiom.2016.11.033 PMC5233814

[46] Ghahremanfard F Mirmohammadkhani M Shahnazari B. The valuable role of measuring serum lipid profile in cancer progression. Oman Med J 2015;30:353–7. 26421116 10.5001/omj.2015.71 PMC4576393

[47] Kumar V Singh A Sidhu DS. A comparitive study to evaluate the role of serum lipid levels in aetiology of carcinoma breast. J Clin Diagn Res 2015;9:C01–3. 10.7860/JCDR/2015/12273.5563 PMC4378769 25859487

[48] Lu CW Lo YH Chen CH. VLDL and LDL, but not HDL, promote breast cancer cell proliferation, metastasis and angiogenesis. Cancer Lett 2017;388:130–8. 27940127 10.1016/j.canlet.2016.11.033

[49] Knapp ML al-Sheibani S Riches PG. Alterations of serum lipids in breast cancer: effects of disease activity, treatment, and hormonal factors. Clin Chem 1991;37:2093–101. 1764785

